# Association of mild cognitive impairment and comorbid depression with postoperative delirium in patients undergoing knee/hip arthroplasty: a prospective cohort study

**DOI:** 10.3389/fmed.2026.1915274

**Published:** 2026-09-22

**Authors:** Xu Lin, Qian He, Kun Wang, Wenjie Kong, Yuanlong Wang, Yizhi Liang, Yanan Lin, Chuan Li, Jiahan Wang, Hongyan Gong, Bin Wang, Fujuan Chen, Yanlin Bi, Yongxin Liang

**Affiliations:** 1 Qingdao University, Qingdao, Shandong, China; 2 Department of Anesthesiology, Qingdao Municipal Hospital, Qingdao, Shandong, China; 3 Department of Anesthesiology, Shandong Second Medical University, Weifang, China; 4 The Second School of Clinical Medicine of Binzhou Medical University, Yantai, China; 5 Department of Emergency Medicine, Qingdao Municipal Hospital, Qingdao, Shandong, China; 6 Peking University People’s Hospital, Women and Children’s Hospital, Qingdao University, Qingdao, Shandong, China

**Keywords:** anesthesiology, cerebrospinal fluid, mild cognitive impairment with comorbid depression, neuropsychological tests, postoperative delirium (POD)

## Abstract

**Background:**

Postoperative delirium (POD) is common after surgery, involving acute disturbances in attention and cognition. While mild cognitive impairment (MCI) and depression each influence cognition, their combined impact on POD is unclear. We aimed to assess the effect of MCI with comorbid depression on POD and explore whether cerebrospinal fluid (CSF) biomarkers mediate this relationship.

**Methods:**

This study included 1,464 knee/hip arthroplasty patients divided into four preoperative diagnostic groups: no disease, MCI only, depression only, and MCI with depression. CSF markers were compared between groups, with subgroup analyses by age/gender. POD risk factors were assessed using multifactorial logistic regression, sensitivity analyses, and mediation effect modeling of CSF markers. A POD prediction model was developed and its performance was evaluated via ROC curve analysis, and three-year postoperative survival differences were analyzed.

**Results:**

POD incidence was 13.9% (*n* = 203). MCI with depression was an independent POD risk factor (OR = 18.56, 95% CI: 8.53–40.77). These patients showed higher T-tau and P-tau, and lower A*β*42 in CSF, with age-related variations. CSF biomarkers partially mediated the association (17.9%–49.9%). The prediction model had an AUC of 0.884 (95% CI: 0.860–0.908). POD patients had significantly lower 3 year survival than non-POD patients, whereas within the POD cohort, MCI with depression was not associated with significantly different mortality.

**Conclusions:**

MCI with comorbid depression is an independent risk factor for POD, and this association is mediated in part by changes in CSF biomarkers.

## Highlights

- Mild cognitive impairment (MCI) with comorbid depression emerged as a strong independent risk factor for postoperative delirium (POD) in knee/hip arthroplasty patients.
- Patients with MCI and depression exhibited distinct CSF biomarker profiles: elevated T-tau and P-tau, reduced A*β*42, and altered A*β*42/T-tau and A*β*42/P-tau ratios compared to controls.
- Age-stratified analysis revealed stronger associations between MCI with depression and CSF biomarkers in older patients (≥65 years), suggesting age-dependent pathophysiological interactions.
- Findings highlight the importance of preoperative screening for cognitive and psychiatric comorbidities to stratify POD risk and guide perioperative interventions.

## Background

Postoperative delirium (POD) is an acute brain dysfunction syndrome characterized by fluctuating changes in attention, cognitive function and level of consciousness (1, 2). POD usually occurs within 1–3 days after surgery, and the clinical manifestations mainly include impaired cognitive function, reduced level of consciousness, attention deficit, psychomotor retardation, and disruption of the sleep-wake cycle (2, 3). POD is associated with poor prognosis, including delayed functional recovery, prolonged intensive care unit (ICU) stays, and increased mortality rates (4). Although the exact pathophysiologic mechanism of POD has not been fully elucidated, existing studies suggest that neuronal injury plays a key role in its pathogenesis, and cerebrospinal fluid (CSF) biomarkers may serve as indicators of neuronal injury (5, 6). Previous studies, including the previous work of our group and other relevant literature reports, have confirmed that the levels of β-amyloid 42 (Aβ42), total tau protein (T-tau), and phosphorylated tau protein (P-tau) in CSF are closely related to the development of POD (7, 8).

Mild cognitive impairment (MCI) is a neurocognitive disorder marked by subtle declines in subjective sensory perception, memory performance, and overall cognitive capacity (9). Depression is a complex condition characterized by persistent sadness, decreased interest or enjoyment in activities that are typically pleasurable, reduced energy levels, challenges in concentration and making decisions, changes in appetite and sleep patterns, altered psychomotor activity, and thoughts of suicide (10). Clinical observations have revealed that depression has a high co-morbidity rate in patients with MCI, with significant overlap in clinical manifestations, both of which can lead to cognitive impairment, decline in daily functioning, and increased risk of death (11, 12).

Previous studies have focused on the separate effects of MCI or depression on the incidence of POD. It has been shown that the risk of POD is significantly higher in patients with MCI than in the cognitively normal population (13), while preoperative depression have been suggested to be an independent risk factor for POD (14, 15). However, to date, no studies have focused on the association between MCI comorbid depression and POD. Recent autopsy studies have shown that more than 50% of MCI patients have mixed pathology, i.e., the presence of both disease-related A*β*/tau lesions and cerebrovascular lesions (16, 17). In addition, reduced A*β*42/T-tau ratio and elevated neurofilament light chain (NfL) were frequently detected in CSF of depressed patients, suggesting that neural axonal damage is associated with depression severity (18, 19). Thus, patients with MCI with comorbid depression may be at increased risk of POD due to this pathologic overlap effect exacerbating postoperative intracerebral microenvironmental disturbances. However, although these studies suggest a possible association between MCI with comorbid depression and POD, there is still a lack of sufficient evidence to support the specific association between the two and its underlying mechanisms, especially the role of CSF biomarkers in it. Therefore, it is clinically important to explore the relationship between MCI comorbid depression and POD and its underlying mechanisms, especially the role of CSF biomarkers in it.

This study was based on a population-based sample and aimed to achieve the following objectives: (1) to clarify the independent effects of MCI with comorbid depression on the occurrence of POD in the Chinese Han population; (2) to compare the differences in CSF biomarkers (A*β*, tau) among patients with MCI with comorbid depression, MCI alone, and depression alone and to analyze the characterization of these differences among different subgroups of the population; (3) to assess the potential mediating role of CSF biomarkers in the association of potential mediating role in the association of MCI with comorbid depression and POD; (4) to construct a risk prediction model for POD; and (5) to analyze three-year postoperative survival differences between POD and non-POD patients, and to explore the impact of different subgroups on survival within the POD cohort.

## Methods

### Eligibility criteria

This prospective cohort study utilized data from Qingdao Municipal Hospital collected between July 2020 and September 2021. Inclusion criteria consisted of: (1) age between 40 and 90 years; (2) scheduled for unilateral knee or hip replacement surgery; (3) ability to independently complete neuropsychiatric assessments; (4) classification as American Society of Anesthesiologists (ASA) grade I–III; and (5) undergoing combined spinal-epidural anesthesia. Exclusion criteria were: (1) a preoperative Mini-Mental State Examination (MMSE) score below 24; (2) history of substance or psychotropic drug abuse or long-term use of steroids or hormonal therapy; (3) significant visual or hearing impairment; (4) severe systemic diseases (e.g., cancer) that may influence cerebrospinal fluid biomarker levels; (5) major psychiatric disorders; or (6) conditions affecting speech or communication. All participants provided written informed consent and were informed of their right to withdraw at any time. The study followed the ethical principles of the Declaration of Helsinki, was approved by the Ethics Committee of Qingdao Municipal Hospital (Ethical Committee 2020 Proceedings Y No. 005), and was registered with the Chinese Clinical Trial Registry (Registration number: ChiCTR2000033439, registration date: 06/07/2020).

### Clinical evaluation and covariates

Before surgery, fundamental patient data were gathered, such as age (continuous), sex (male vs. female), years of education (continuous), MMSE score (continuous), body mass index (BMI: continuous), smoking status (nonsmoker vs. current/former smoker), and alcohol consumption (nonsmoker vs. drinker). Moreover, details regarding patients' medical histories and existing comorbid conditions were obtained from medical documentation.

In this study, initial analyses assessed 13 covariates that were subsequently included in logistic regression modeling. According to earlier research, POD occurred at a rate of approximately 10%, with an anticipated loss-to-follow-up rate of 20%. Using the logistic regression method for calculating sample size based on events per variable (EPV), the EPV was determined as 10 (20). Consequently, the calculated necessary sample size was 1,625 participants (computed as 13 × 10 ÷ 0.10 ÷ 0.8 = 1,625). Duration of surgery, duration of anesthesia, and estimated blood loss were prospectively recorded. As these variables showed no significant intergroup differences (Table 1) and did not affect the primary effect estimates, they were not included in the final regression models.

**Table 1:** Demographic and characteristics of participants.

Characteristics	NPOD	POD	*P*
Number	1,261	203	
Age, M (IQR), years	62 (54–67)	74 (71–78)	<0.001***
BMI, M (IQR)	25.40 (23.04–27.40)	25.30 (23.00–27.70)	0.691
Education, M (IQR), year	9 (8–12)	9 (6–12)	0.938
MMES, score, M (IQR)	28 (27–29)	28 (27–29)	0.016*
Duration of surgery, M (IQR), h	1.83 (1.66–2.33)	2.00 (1.83–2.17)	0.826
Duration of anesthesia, M (IQR), h	2.33 (1.66–2.5)	2.33 (2.17–2.67)	0.697
Estimated blood loss, M (IQR), mL	20 (5–200)	30 (10–200)	0.672
Sex(female/male)	707/552	121/82	0.557
Hypertension, yes (%)	439 (34.8)	95 (46.8)	0.001**
Diabetes, yes (%)	191 (15.2)	56 (27.6)	<0.001***
CHD, yes (%)	140 (11.1)	52 (25.6)	<0.001***
Cigarette smoking, yes (%)	372 (29.5)	54 (26.6)	0.395
Drinking, yes (%)	453 (36.0)	57 (28.1)	0.027*
MCI, yes (%)	257 (20.4)	117 (57.6)	<0.001***
Depression, yes (%)	41 (3.3)	35 (17.2)	<0.001***
MCI with comorbid depression, yes (%)	9 (0.7)	24 (11.8)	<0.001***
CSF biomarkers, M (IQR), pg/mL			
Aβ42	407.638 (264.581–594.400)	270.100 (152.768–418.012)	<0.001***
T-tau	187.800 (140.750–256.564)	271.624 (193.808–596.4001)	<0.001***
P-tau	39.482 (31.295–50.295)	56.510 (39.688–78.486)	<0.001***
Ratios of biomarkers, M (IQR)			
Aβ42/T-tau	2.188 (1.316–3.254)	1.073 (0.445–1.772)	<0.001***
Aβ42/P-tau	10.240 (6.326–14.846)	5.115 (2.211–8.682)	<0.001***

Categorical variables are reported as numbers and percentages; continuous variables are reported as means ± SD, whereas non-normal data are expressed as the M (Q25, Q75).

POD, Postoperative delirium; NPOD, No postoperative delirium; M, Median; IQR, Interquartile Range; BMI, Body mass index; MMSE, Minimum mental state examination; CHD, Coronary heart disease; MCI, Mild cognitive impairment; CSF, Cerebrospinal fluid.

* *P*-Value < 0.05.

** *P*-Value < 0.01.

*** *P*-Value < 0.001.

### MCI diagnostic criteria

In this study, the Winblad criteria were used for the diagnosis of MCI (21). Based on age- and education-adjusted normative data, cognitive decline was defined as a decrease in scores on objective neuropsychological tests of at least 1.5 standard deviations (SDs) from premorbid levels. Specifically, the following criteria were required for the diagnosis of MCI: (1) Both subjective and objective cognitive decline were present. Subjective cognitive decline was defined as cognitive decline reported by the patient or their relatives, whereas objective cognitive decline was defined as cognitive functioning that is significantly below expected levels as assessed by standardized neuropsychological tests. (2) Cognitive decline was not sufficient to interfere with activities of daily living. Although patients showed significant decline in some cognitive domains, they were able to maintain general functioning (i.e., minimal, if any, change in basic and complex activities of daily living). In addition, the diagnosis of MCI was made in conjunction with the patient's medical history, physical examination, and imaging findings. In this study, all patients with MCI were evaluated by a specialized neurologist, and other potential causes of cognitive impairment were excluded.

### Assessment of depressive symptoms

Depressive symptoms were assessed using the 17-item Hamilton Depression Rating Scale (HAMD), which consists of 17 items each scored 0–4 (total range 0–52) (22). A total score of ≥8 was used to define the presence of clinically relevant depressive symptoms; for brevity, this condition is referred to as “depression” in the manuscript, though it does not equate to a clinical diagnosis of major depressive disorder.

Study participants were assessed for HAMD by a professionally trained assessor. The assessment process followed the standard operating procedures of the HAMD, and relevant information about the participants' mood, cognition, behavior, and somatic symptoms was collected through observation and interview. The evaluator rated each item based on the participant's responses and performance and ultimately calculated a total HAMD score. In addition, the final diagnosis was made by combining the clinical interview and other relevant information to ensure the accuracy and reliability of the diagnosis.

### Anesthesia and surgery

Patients were given spinal anesthesia combined with epidural anesthesia before undergoing hip or knee replacement surgery. Prior to surgery, patients were required to fast and abstain from food and drink for 6–8 h. Immediately after entering the operating room, routine monitoring was performed, including electrocardiogram, oxygen saturation, arterial blood pressure, and establishment of intravenous access.

A lumbar puncture was performed at the L3–L4 vertebral space, and 2 mL of CSF was withdrawn for biomarker testing. Subsequently, spinal anesthesia was induced by slow injection of 2–2.5 mL of 0.66% ropivacaine over 30 s. All patients received intravenous remimazolam for intraoperative sedation at 0.05 mg kg⁻¹ h⁻¹ following the recommended protocol. During the procedure, the sensory block level was maintained below T8, and vital signs (blood pressure, heart rate, oxygen saturation) were monitored every 3 min. Oxygen was supplied via face mask at 5 L/min throughout the procedure. Postoperatively, the patient was observed in the anesthesia recovery unit (PACU) for 30 min and was transferred back to the general ward after stabilization.

### Collection and measurement of CSF biomarkers

A volume of 2 mL of CSF was gathered in a polypropylene centrifuge tube. Subsequently, the sample underwent centrifugation at 2,000 × g for a duration of 10 min under room—temperature conditions. After centrifugation, the supernatant was carefully decanted into an enzyme—free Eppendorf tube (specifically a PCR02—C oxygen bottle). Finally, this supernatant was stored at a temperature of −80 °C for the purpose of subsequent analyses (23).

Enzyme-linked immunosorbent assay (ELISA) was employed to quantitatively determine the concentrations of A*β*42, T-tau, and P-tau in 2 mL of CSF, using commercially available assay kits (Thermo Scientific, Multiskan MK3) according to the manufacturer's protocols. The ratios of A*β*42/T-tau and A*β*42/P-tau were subsequently calculated. All measurements were conducted by the same laboratory personnel with the group assignments blinded to ensure objectivity.

### Neuropsychological testing

Prior to surgery, cognitive function was evaluated through the MMSE with an exclusion threshold of scores <24. Postoperative monitoring for delirium was systematically implemented through twice-daily assessments (9:00–10:00 and 14:00–15:00) from postoperative day 1 until discharge or day 7, whichever occurred first. A board-certified anesthesiologist administered these evaluations using the Confusion Assessment Method (CAM) protocol. Participants meeting CAM diagnostic criteria were stratified into the POD cohort, whereas those maintaining normal cognitive profiles formed the non-POD (NPOD) comparison group. This stratification protocol was maintained throughout the 7-day observation window or until hospital discharge. Both assessment instruments—the MMSE for baseline cognition and CAM for delirium detection—represent gold-standard validated tools in neurocognitive evaluation (24, 25).

### Follow-up

All participants were scheduled for a standardized 36-month postoperative follow-up. Vital status was verified through structured follow-up protocols, including outpatient clinic visits and regular structured telephone interviews. Loss to follow-up was defined as failure to contact the participant or their family members after at least three consecutive attempts via telephone or clinic visit. The date and cause of death, if occurred, were documented from hospital records or death certificates.

### Statistical analysis

In this study, the Kolmogorov–Smirnov test was used to assess whether the data distribution of continuous variables conformed to normality. Mean ± standard deviation (Mean ± SD) was used for descriptive statistics for variables that conformed to normal distribution, while median and interquartile range (IQR [Q25, Q75]) were used for variables that did not conform to normal distribution. Depending on the distributional characteristics of the data, the analysis of variance of continuous variables between groups was performed using the independent samples *t*-test (for normal distribution) or the Mann–Whitney *U*-test (for non-normal distribution), respectively. Comparisons between groups for categorical variables were performed using the chi-square test (*χ*² test) or Fisher's exact test. To optimize the data distribution of CSF biomarkers and to eliminate the effect of magnitude, Box-Cox transformations were first performed, followed by standardization of Z-scores. Differences between groups were assessed by one-way analysis of variance (ANOVA), and Tukey's HSD *post hoc* test was used for pairwise comparisons of significant differences.

Binary Logistic regression models were used to assess the association between POD and MCI, depression, MCI comorbid depression, and CSF biomarkers. The multivariate regression model incorporated baseline indicators with adjustments for established demographic factors (age, sex, education years, BMI) and clinical baseline comorbidities (e.g., diabetes mellitus). Duration of surgery, duration of anesthesia, and estimated blood loss were prospectively recorded. Given that all participants underwent standardized elective unilateral arthroplasty, these procedural parameters showed no significant intergroup differences (Table 1). Thus, after considering model parsimony, they were not retained in the final multivariable models, which were constructed with adjustment for established demographic factors (age, sex, education years, BMI) and clinical baseline comorbidities (e.g., diabetes mellitus). To assess the risk of potential multicollinearity, variance inflation factor (VIF) analysis was performed to test the degree of covariance between variables. Sensitivity analyses were conducted to evaluate the robustness of the findings. Subsequently, CSF biomarker levels were compared between the MCI- and depression-free controls group, the MCI group, the depression group, and the MCI with comorbid depression group. In addition, multivariate linear regression (MLR) analyses were performed with age (middle-aged group: <65 years; elderly group: ≥65 years) and gender (female, male) as stratification variables, aiming to explore the associations between CSF biomarkers and MCI, depression, and MCI with comorbid depression across age and gender groups, and the results of the study were visualized by heatmaps.

Further, this study examined the mediating effect of CSF biomarkers in the association of MCI comorbid depression with POD using the Bootstrap method. To mitigate potential multicollinearity issues, CSF biomarkers [A*β*42, T-tau, P-tau, A*β*42/T-tau, A*β*42/P-tau] were centered before analysis. Specifically, five independent simple mediation models were constructed with MCI comorbid depression as the independent variable, POD as the dependent variable, and each centered CSF biomarker as the mediator variable. The mediating effects were assessed according to the following criteria: (1) the strength of the association between MCI with comorbid depression and POD was attenuated with the inclusion of CSF biomarkers in the model; (2) there was a significant correlation between CSF biomarkers and POD; (3) there was a significant correlation between MCI with comorbid depression and POD; and (4) there was a significant correlation between MCI with comorbid depression and CSF biomarkers.

Subsequently, a multivariate Logistic regression clinical prediction model was constructed to identify people at high risk for POD.

For survival analysis, overall survival time was defined as the interval from the date of surgery to the date of death from any cause. For participants who were lost to follow-up or remained alive at the end of the 36-month observation period, survival time was censored at their last confirmed contact date or at 36 months, respectively. Kaplan–Meier survival curves were constructed to compare postoperative mortality between POD and non-POD groups, as well as among POD subgroups. Differences in survival distributions were assessed using the log-rank test.

Data were analyzed using SPSS 25.0 (IBM SPSS, Chicago, USA), R software 4.3.1 (R Foundation for Statistical Computing, Vienna, Austria) and Stata MP16.0 (Solvu Soft, Chicago, USA). The significance criterion was *P* < 0.05.

### Statement

The work has been reported in line with the STROCSS criteria (26).

## Results

### Patient screening and grouping

Of the 1,625 patients initially screened, 161 were excluded according to the exclusion criteria, and 1,464 patients were finally included in this study with complete clinical data. The patients were categorized into the POD group (203 cases) and the non-POD group (NPOD group, 1,261 cases) according to whether POD occurred after surgery. Figure 1 outlines how patients were screened, with 161 excluded based on predefined criteria.

**Figure 1:**
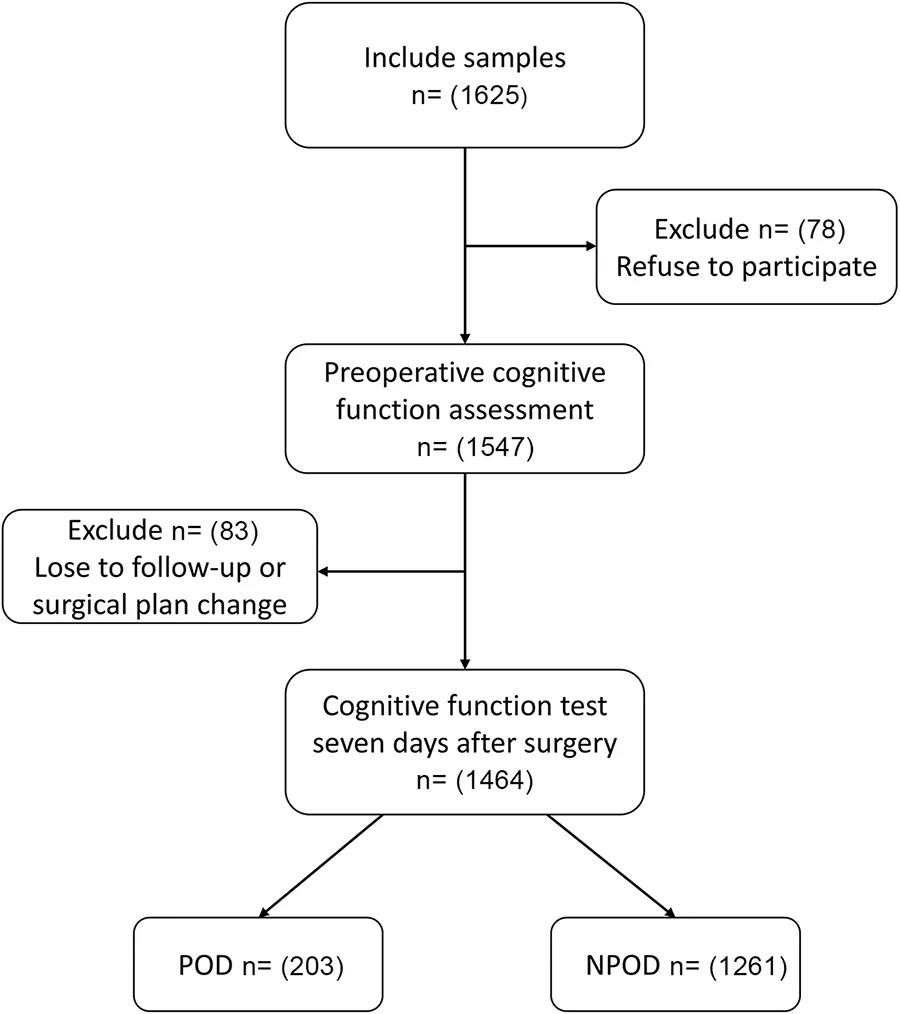
The flow diagram showed the selection of eligible patients and the enrollment process. POD, Postoperative delirium; NPOD, No postoperative delirium.

### Clinical characteristics of the patients

Of the 1,464 patients undergoing surgical anesthesia, POD occurred in 203, and the overall incidence of POD was 13.9%.

Compared with patients in the NPOD group, patients in the POD group had the following characteristics: significantly older age (*P* < 0.001), significantly lower scores on the Brief Mental State Evaluation Scale (MMSE) (*P* = 0.041), and a lower percentage of alcohol consumption (*P* = 0.027). In addition, patients in the POD group had a significantly higher prevalence of hypertension (*P* = 0.001), diabetes mellitus (*P* < 0.001), and coronary heart disease (CHD) (*P* < 0.001), as well as a significantly higher prevalence of MCI, depression, and MCI with comorbid depression than the NPOD group (*P* < 0.001).

In terms of CSF biomarkers, there was a significant difference in the levels of A*β*42, T-tau, P-tau, A*β*42/T-tau, and A*β*42/P-tau between patients in the POD group and those in the NPOD group (*P* < 0.001). Specifically, A*β*42 levels were significantly lower, while T-tau and P-tau levels were significantly higher in patients in the POD group.

However, there were no significant differences between the two groups in terms of BMI, years of education, sex ratio and smoking history (all *P* > 0.05). Similarly, no significant differences were observed in duration of surgery, duration of anesthesia, or estimated blood loss between the groups (all *P* > 0.05). Detailed clinical characteristics of the patients are shown in Table 1.

### Logistic regression of risk and protective factors for POD

In this study, risk factors for POD were first screened by univariate logistic regression analysis, which showed that MCI (OR = 5.315, 95% CI: 3.897–7.248), depression (OR = 6.199, 95% CI: 3.840–10.009), MCI with comorbid depression (OR = 18.562, 95% CI: 8.534–40.767), as well as elevated CSF biomarkers T-tau (OR = 1.006, 95% CI: 1.005–1.007) and P-tau (OR = 1.049, 95% CI: 1.041–1.058), were significantly associated with increased odds of POD (all *P* < 0.001). Conversely, reductions in A*β*42 (OR = 0.997, 95% CI: 0.996–0.998), A*β*42/T-tau (OR = 0.418, 95% CI: 0.353–0.496), and A*β*42/P-tau (OR = 0.847, 95% CI: 0.817–0.878) were associated with decreased odds of POD (all *P* < 0.001). In addition, we constructed a multivariate logistic regression in which the above associations remained statistically significant with consistent directions of effect; sensitivity analyses further verified the stability of the results (see Table 2 for details). The covariance diagnostic showed that the VIF for all variables was <5, suggesting no significant multicollinearity (Supplementary Table S1) (27). To address sparse-data bias for the MCI with comorbid depression subgroup, Firth's penalized maximum likelihood estimation was performed across all three logistic models (Supplementary Table S2). The Firth-corrected ORs remained significant across all models (Model 3: OR = 8.455, 95% CI: 3.483–22.167, *P* < 0.001), confirming the robustness of the association between MCI with comorbid depression and POD.

**Table 2:** Logistic analysis for risk factors of POD patients.

Factors of interest	Model 1		Model 2		Model 3	
	OR (95% CI)	*P*-value	OR (95% CI)	*P*-value	OR (95% CI)	*P*-value
MCI	5.315 (3.897–7.248)	<0.001***	4.015 (2.808–5.739)	<0.001***	4.394 (3.020–6.392)	<0.001***
Depression	6.199 (3.840–10.009)	<0.001***	3.475 (1.944–6.213)	<0.001***	3.085 (1.661–5.728)	<0.001***
MCI with comorbid depression	18.562 (8.534–40.767)	<0.001***	9.184 (3.718–22.686)	<0.001***	9.032 (3.484–23.416)	<0.001***
Aβ42	0.997 (0.996–0.998)	<0.001***	0.997 (0.996–0.998)	<0.001***	0.997 (0.996–0.998)	<0.001***
T-tau	1.006 (1.005–1.007)	<0.001***	1.005 (1.004–1.006)	<0.001***	1.005 (1.004–1.007)	<0.001***
P-tau	1.049 (1.041–1.058)	<0.001***	1.039 (1.030–1.048)	<0.001***	1.039 (1.030–1.049)	<0.001***
Aβ42/T-tau	0.418 (0.353–0.496)	<0.001***	0.468 (0.388–0.564)	<0.001***	0.471 (0.389–0.570)	<0.001***
Aβ42/P-tau	0.847 (0.817–0.878)	<0.001***	0.870 (0.839–0.903)	<0.001***	0.874 (0.842–0.907)	<0.001***

OR, odds ratio; 95% CI, 95% confidence interval; MCI, Mild cognitive impairment.

Model 1: the unadjusted logistic regression.

Model 2: adjusted logistic regression.

Model 3: sensitivity analysis was based on more covariates including hypertension, drinking, CHD, cigarette smoking and MMSE.

*** *P*-Value < 0.001.

### Comparison of CSF markers between groups

Comparison of CSF marker levels between the groups showed that A*β*42 levels were significantly lower in the MCI with comorbid depression group compared to the control and MCI groups, but not significantly different from the depression group; T-tau levels were significantly higher in the MCI with comorbid depression group compared to the control and MCI groups, but not significantly different from the depression group; and P-tau levels were significantly higher in the MCI with comorbid depression group compared to the control, MCI and depression groups (Figure 2).

**Figure 2:**
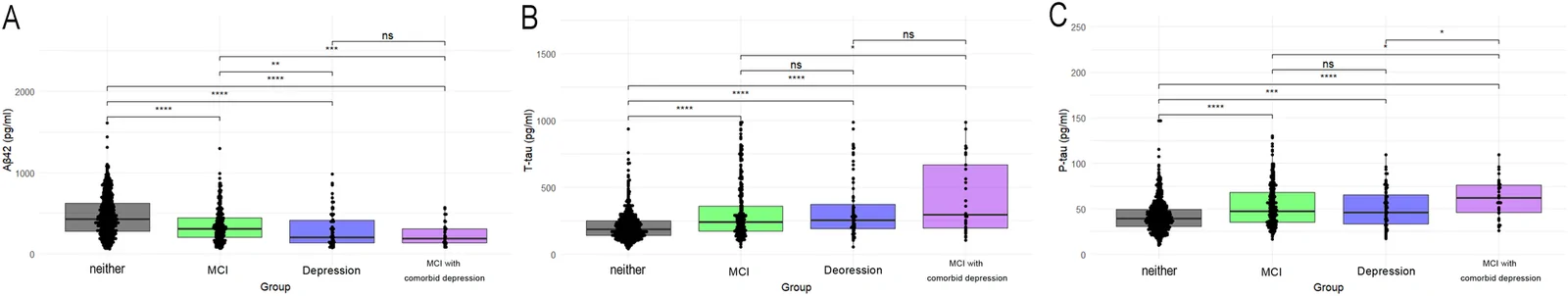
CSF-related biomarkers across the four different groups in panels **(A)** A*β*42, **(B)** T-tau, and **(C)** P-tau. Bars indicate mean values and error bars indicate standard deviation. Swarm plots show the distribution of the biomarker data. *z*-score normalized by Box-Cox power transformation for CSF-related biomarkers. One-way ANOVA was used to test for between-group differences in biomarkers, followed by Tukey HSD *post hoc* tests. *P* > 0.05: NS; *P* ≤ 0.05: *; *P* ≤ 0.01: **; *P* ≤ 0.001: ***; *P* ≤ 0.0001: ****. MCI, Mild cognitive impairment; CSF, cerebrospinal fluid.

### Interaction and stratified analysis by age and sex

Interaction analyses indicated that age was a key factor moderating the association between MCI with comorbid depression and CSF biomarkers. Specifically, the association between MCI with comorbid depression and CSF biomarkers was generally non-significant in the middle-aged group; whereas it was significantly stronger in the older age group. The heatmap (Figure 3) clearly demonstrates this age difference: the MCI with comorbid depression and depression subgroups in the older age group showed more pronounced differences (darker colors) in both P-tau and T-tau levels, reflecting stronger statistical significance. In addition, stratified analyses further revealed age- and gender-specific patterns of biomarker associations: the association of MCI with P-tau was more prominent in the older age group; whereas the association of depression with P-tau was more pronounced in males; and the association of depression with T-tau was likewise more pronounced in the older age group.

**Figure 3:**
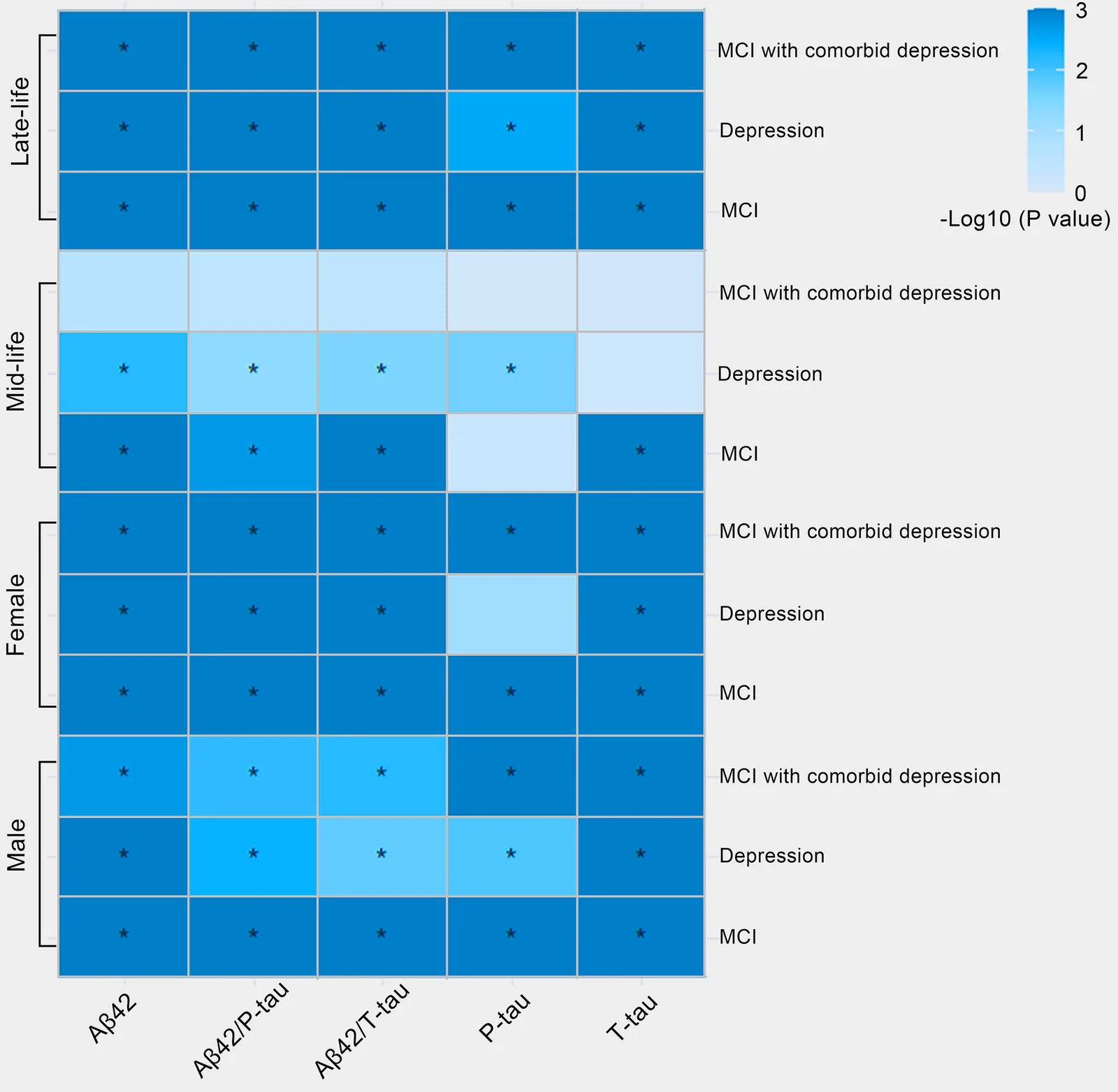
Stratified analyses of associations between CSF AD biomarkers and MCI with comorbid depression. Multiple linear regression models were employed with adjustment for age, gender, BMI, years of education, diabetes, hypertension, drinking, CHD, cigarette smoking and MMSE. Stratified analyses showed that MCI with comorbid depression was significantly associated with CSF biomarkers (T-tau, P-tau, A*β*42, and A*β*42/tau ratio) in older (≥65 years old) patients but not in middle-aged (<65 years old) patients. POD, Postoperative delirium; MCI, Mild cognitive impairment.

### Mediation analysis

It was found that the relationship between MCI combined with depression and POD was mainly mediated by T-tau, P-tau, A*β*42/T-tau ratio and A*β*42/P-tau ratio. The proportion of mediated effects ranged from 17.9% to 49.9%, as shown in Figure 4.

**Figure 4:**
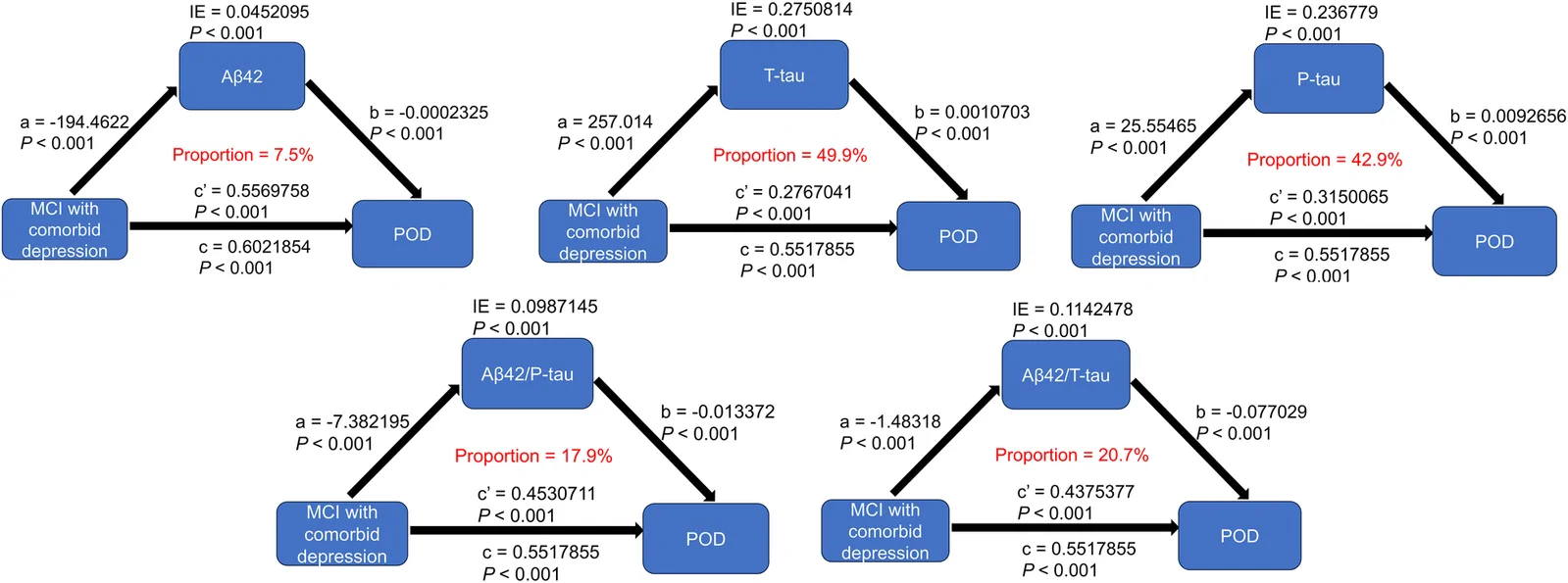
Mediation analyses with 10,000 bootstrapped iterations were used to examine the mediation effects of Ab42, T-tau, P-tau, Ab42/T-tau, and Ab42/P-tau on POD. POD, Postoperative delirium; MCI, Mild cognitive impairment.

### Construction of a clinical prediction model for POD based on perioperative risk factors

A multivariate logistic clinical prediction model constructed using age, gender, MMSE score, hypertension, diabetes, CHD, history of alcohol consumption, and MCI with comorbid depression had the highest predictive value for the incidence of POD.

In this study, subject work characteristics (ROC) curves were plotted to assess the classification performance of the model. As shown in the figure, the ROC curve as a whole lies above the diagonal (randomized classification benchmark), indicating that the model has good discriminatory ability. The area under the curve (AUC) is 0.884 (95% CI: 0.860–0.908), indicating that the model has superior classification performance. In addition, the optimal threshold labeled in the figure corresponds to a false-positive rate (1 − specificity) of about 0.152, which corresponds to a sensitivity of 0.847 and a specificity of 0.817. This result indicates that the model is able to achieve good sensitivity while maintaining high specificity at this threshold. Decision curve analysis (DCA) showed that the model exhibited high standardized net returns at all risk thresholds, especially at moderate to high risk thresholds, where it significantly outperformed the “treat all” or “treat none” strategy models (Figure 5).

**Figure 5:**
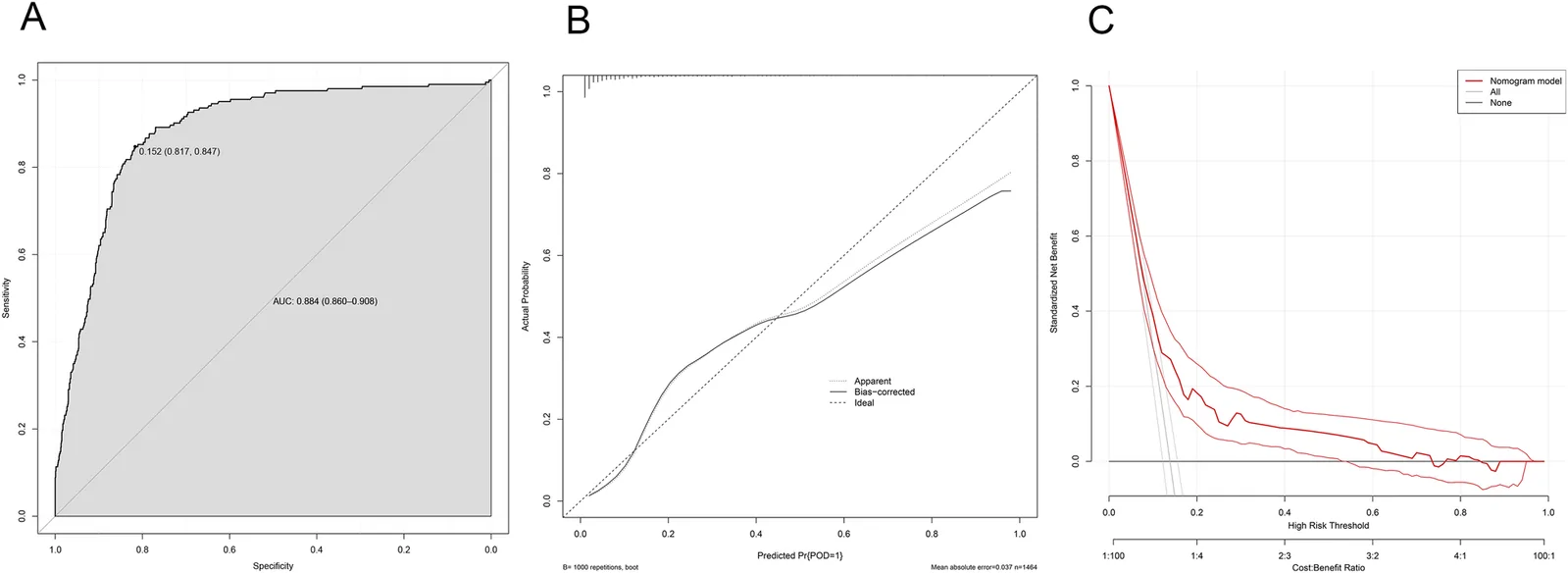
**(A)** Receiver operating characteristic curve of the predictive probabilities obtained from the multivariate logistic clinical prediction model. **(B)** Calibration plot. **(C)** Decision curve analysis.

### Survival analysis

During the 36-month follow-up, 29 deaths (14.3%) and 20 losses (9.9%) were recorded in the POD group, compared with 58 deaths (4.6%) and 126 losses (10.0%) in the non-POD group. Kaplan–Meier analysis with right-censoring showed that POD patients had significantly lower 3-year survival than non-POD patients (*P* < 0.001; Figure 6A). Within the POD cohort, stratification by preoperative cognitive/psychiatric status (neither, MCI only, depression only, MCI with comorbid depression) revealed no significant difference in 3-year survival after censoring correction (*P* = 0.79; Figure 6B), indicating that while POD itself conferred a significant long-term mortality risk, the specific MCI/depression subtypes did not further stratify prognosis among patients who already developed POD.

**Figure 6:**
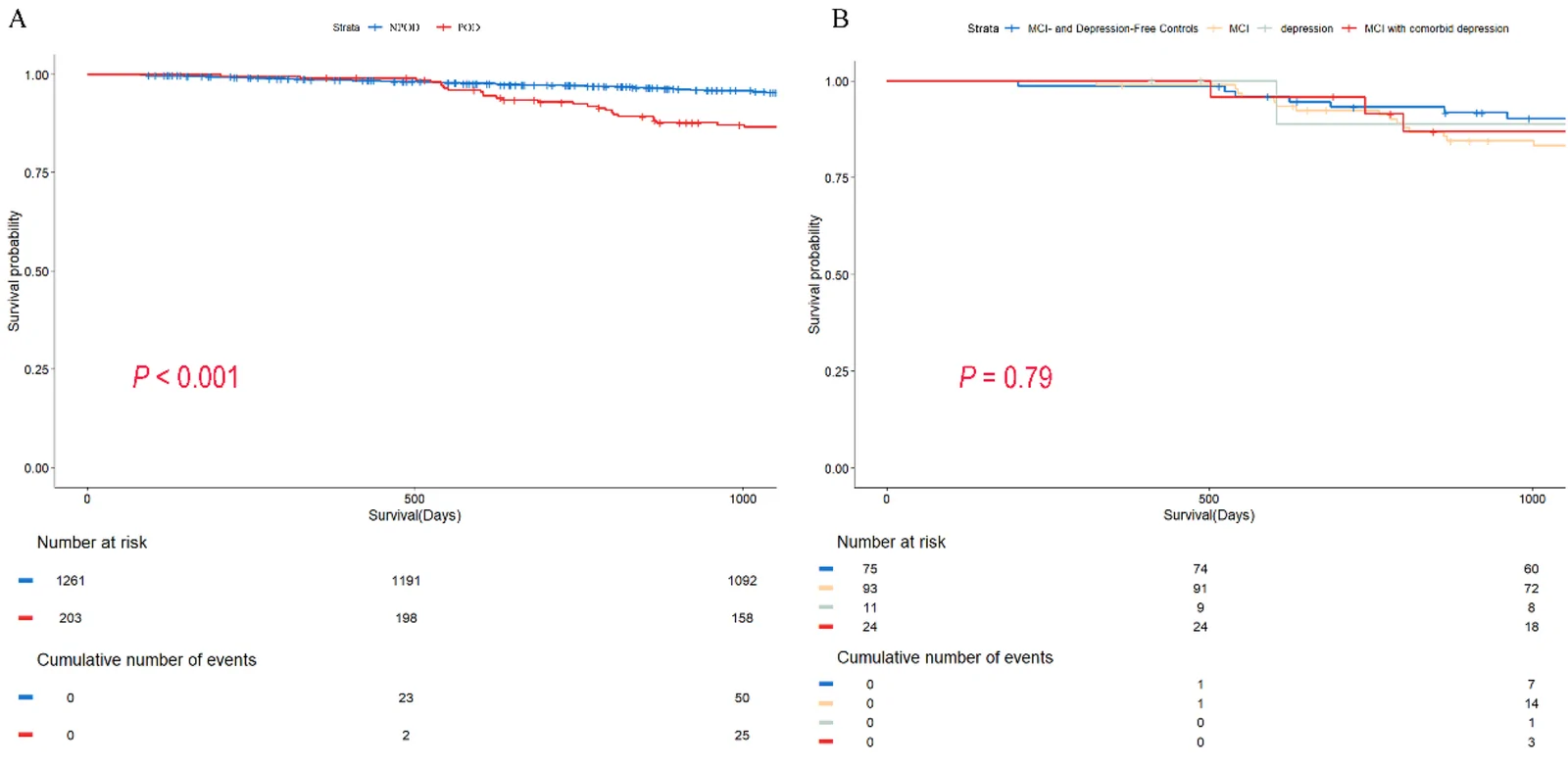
Kaplan–Meier survival curves for 3-year overall survival after surgery. **(A)** Comparison between POD and non-POD groups. **(B)** Survival analysis of POD patients stratified by preoperative MCI and depression status. POD, Postoperative delirium; MCI, Mild cognitive impairment.

## Discussion

This study is the first to demonstrate that MCI with comorbid depression is independently associated with POD risk, in which T-tau, P-tau, A*β*42/T-tau ratio, and A*β*42/P-tau ratio play a mediating effect, and the amounts of these mediators are correlated by age and gender. In addition, MCI with comorbid depression did not increase three-year postoperative mortality in POD patients.

For the first time, we identified MCI with comorbid depression as an independent risk factor for POD. Previously, it was shown that a single MCI can increase the risk of POD by affecting amyloid and tau proteins (18). And, it has also been shown that increased severity of depressive symptoms is negatively correlated with CSF A*β*42/T-tau and A*β*42/P-tau ratios and is an independent risk factor for POD (19). MCI is more common in patients with depression, with neuropathology showing an underlying cerebrovascular etiology for depression, and this combination of cerebrovascular changes and neuropathologic changes in CSF markers is the most common cause of cognitive impairment in older adults (28, 29). In addition, MCI cases with this “mixed” pathology are more likely to turn into dementia than MCI cases with “pure CSF marker neuropathologic” changes (30). However, no one has previously explored whether MCI with comorbid depression increases the risk of POD, and our study fills this gap and links CSF markers to explore the specific relationship between these.

Our study demonstrated that elevated A*β*42 is a protective factor for POD and elevated T-tau protein and P-tau protein are risk factors for POD, which is in line with previous studies (31–33). Under normal conditions, only trace amounts of A*β* are detected in brain tissue. Under pathological conditions, however, impaired clearance of extracellular A*β* leads to its accumulation, which in turn stimulates cytokine production and promotes the conversion of soluble A*β* into insoluble filaments. These filaments deposit both intra- and extracellularly, ultimately contributing to neuronal injury, neurodegeneration, and cognitive impairment (34, 35). Tau is a microtubule-associated protein involved in microtubule dynamics that functions as a track for axonal transport (36). In cognitive decline, tau-related pathology begins with the granular accumulation of P-tau in the cytoplasm, axons, and dendrites. Then, tau gradually accumulates to form neurofilaments and neurogenic fiber tangles, which affect neuronal processes that ultimately degenerate (37, 38). In addition, our results demonstrate that MCI with comorbid depression is more pronounced than single MCI or depression changes in biological CSF biomarkers, which may be attributable to the combined burden of MCI and depressive symptoms on neurological pathways. Large autopsy series provide evidence that MCI is often associated with cardiovascular disease, especially cerebral microarteriosclerosis (30). And severe cerebral microarteriosclerosis is associated with overall cognitive impairment (39, 40). Thus, the superimposed effect of MCI with comorbid depression in terms of neurologic damage may be related to the superimposed cerebrovascular damage.

Although both amyloid and tau protein lesions characterize POD, our results suggest that the link between MCI with comorbid depression and POD is mediated primarily by tau proteins, but the exact mechanism is unknown to us. Previously, it has been shown that tau protein lesions are more strongly associated with cognitive decline than amyloid lesions (41). Whereas dynamic interactions between neurons, glial cells, and vascular cells contribute to the maintenance of homeostasis in the brain microenvironment (42). Therefore, we hypothesized that cerebrovascular damage associated with MCI comorbid depression may contribute to an imbalance in the brain microenvironment, potentially influencing cerebral blood flow and thereby contributing to inadequate cerebral perfusion. These changes may exacerbate tau pathology by disrupting neurons, neuron-glia interactions, and the blood-brain barrier.

The analytical findings revealed a significantly stronger association between MCI with comorbid depression and CSF biomarkers in the elderly subgroup compared to younger counterparts. Previous studies have shown that advanced age itself is a risk factor for POD and can influence CSF biomarkers (7). Age-related processes, including atherosclerosis, inflammation, and endocrine and immune changes, compromise the integrity of the prefrontal-striatal pathway, the amygdala, and the hippocampus, factors that increase the vulnerability of the organism. It has been reported in the literature that depression primarily affects those with chronic medical conditions and cognitive impairments in the elderly population and worsens the outcome of many diseases (43, 44). Previous studies have suggested that younger populations may experience a higher prevalence of depressive symptoms due to psychosocial stressors associated with career and family development; however, the impact of these symptoms on cognitive function is weaker in younger than in older populations (45, 46). Moreover, older patients are more likely to have MCI cases with “mixed” pathology. These also explain why patients with depression alone and MCI alone have stronger associations with individual CSF markers in the elderly population. This observation suggests that further stratification of age-dependent pathophysiologic mechanisms is warranted, particularly regarding the synergistic role of neurodegenerative and vascular pathologies in MCI with comorbid depression.

There are limited treatment options for POD, so preoperative identification of high-risk individuals and timely prevention are important tools to reduce the incidence of POD (47, 48). In addition to MCI with comorbid depression, the present study also found that hypertension, diabetes, lower MMSE scores, and other risk factors were significantly associated with the incidence of POD. These findings underscore the clinical utility of preoperative risk stratification incorporating MCI with comorbid depression and other readily measurable risk factors, providing stronger evidence-based support for preoperative risk stratification. Optimized perioperative management of modifiable risk factors may reduce the incidence of POD and potentially improve both short- and long-term patient outcomes.

It is important to clarify the terminology of perioperative neurocognitive disorders. POD, assessed by CAM in this study, is an acute, fluctuating disturbance of attention occurring in the immediate postoperative period (49). Under the updated nomenclature of perioperative neurocognitive disorders (PND) (50), POD should be conceptually distinguished from longer-term cognitive decline.

In contrast to the non-POD group, POD patients exhibited significantly higher 3-year mortality, underscoring the long-term prognostic burden of delirium. However, among POD patients, those with MCI and comorbid depression did not show a significantly increased mortality risk compared with other POD subgroups. This finding differs from some of the reports in the literature. Previous studies have shown that depression is associated with vascular endothelial dysfunction and increases cardiovascular mortality (51, 52). In addition, depression or apathy in patients with cognitive dysfunction may exacerbate chronic disease management difficulties and accelerate cognitive and functional progression, ultimately leading to an increased risk of death (53). One possible explanation is that the survival analyses in this study were performed on patients who had developed POD and had poorer overall health at baseline, which may have attenuated the observed associations between MCI with comorbid depression and long-term prognosis. However, further studies are needed to explain this discrepancy.

Several limitations of this study should be acknowledged. First, MCI and depression were treated as categorical variables without incorporating severity scales, which may have attenuated the strength of the observed associations. Second, the study cohort was restricted to the Chinese Han population, which limits the generalizability of our findings to other ethnic or geographic groups. Third, excluding patients with baseline MMSE scores below 24 limits the generalizability of our prediction model. As individuals with lower baseline cognitive function are at inherently higher risk for POD, this restriction may underestimate overall POD incidence. Consequently, our model is primarily applicable to non-demented surgical patients or those with mild cognitive impairment, rather than moderate-to-severe dementia. Future studies with broader baseline cognitive spectra are warranted to validate and extend our model.

## Conclusion

In conclusion, this prospective cohort study indicates that MCI with comorbid depression is an independent risk factor for POD, which is particularly evident in elderly patients. Our findings also suggest that tau protein plays an important mediating role between MCI with comorbid depression and POD. Based on the risk factors of POD patients, we developed a prediction model, which provides a reference basis for clinicians to identify high-risk patients at an early stage. Notably, MCI with comorbid depression did not increase the three-year postoperative mortality rate of POD patients.

## Data Availability

The raw data supporting the conclusions of this article will be made available by the authors, without undue reservation.
