# Supplementary material for: Association of mild cognitive impairment and comorbid depression with postoperative delirium in patients undergoing knee/hip arthroplasty: a prospective cohort study
Source: Front Med (Lausanne). 2026 Sep 22;13:1915274. doi: 10.3389/fmed.2026.1915274 (PMC13638664; doi:10.3389/fmed.2026.1915274)
Supplement: Supplementary file 1 [file Table1.docx]

**Supplementary table 1.** covariance analysis

|  | | *P* | collinearity check | |  |
| --- | --- | --- | --- | --- | --- |
|  |  |  | tolerances | VIF |  |
|  | MCI with comorbid depression | 0.016 | 0.537 | 1.861 |  |
|  | Depression | 0.139 | 0.557 | 1.795 |  |
|  | MCI | 0.002 | 0.774 | 1.292 |  |
|  | Aβ42 | <0.001 | 0.233 | 4.287 |  |
|  | T-tau | <0.001 | 0.504 | 1.983 |  |
|  | P-tau | <0.001 | 0.395 | 2.534 |  |
|  | Aβ42/T-tau | 0.034 | 0.329 | 3.040 |  |
|  | Aβ42/P-tau | 0.013 | 0.206 | 4.845 |  |
|  | Age | <0.001 | 0.687 | 1.455 |  |
|  | Sex | 0.547 | 0.630 | 1.587 |  |
|  | MMSE | <0.001 | 0.836 | 1.196 |  |
|  | Education years | 0.991 | 0.987 | 1.013 |  |
|  | alcohol history | 0.089 | 0.649 | 1.540 |  |
|  | smoking history | 0.886 | 0.605 | 1.654 |  |
|  | Diabetes | 0.408 | 0.904 | 1.106 |  |
|  | Hypertension | 0.411 | 0.856 | 1.168 |  |
|  | CHD | 0.307 | 0.869 | 1.151 |  |
|  | BMI | 0.954 | 0.936 | 1.068 |  |
| a. implicit variable：POD | | | | | |
