# Supplementary material for: Association of mild cognitive impairment and comorbid depression with postoperative delirium in patients undergoing knee/hip arthroplasty: a prospective cohort study
Source: Front Med (Lausanne). 2026 Sep 22;13:1915274. doi: 10.3389/fmed.2026.1915274 (PMC13638664; doi:10.3389/fmed.2026.1915274)
Supplement: Supplementary file 2 [file Table2.docx]

**Supplementary Table 2** Firth‑corrected ORs for MCI with comorbid depression and POD

| Model | Original OR (95% CI) | Firth-corrected OR (95% CI) |
| --- | --- | --- |
| Model 1 | 18.562 (8.534–40.767) | 16.268 (7.969–35.541) |
| Model 2 | 9.184 (3.718–22.686) | 7.394 (3.225–18.149) |
| Model 3 | 9.032 (3.484–23.416) | 8.455 (3.483 – 22.167) |

Model 1: the unadjusted logistic regression.

Model 2: adjusted logistic regression.

Model 3: sensitivity analysis was based on more covariates including hypertension, drinking, CHD, cigarette smoking and MMSE.
